# Tectal glioma as a distinct diagnostic entity: a comprehensive clinical, imaging, histologic and molecular analysis

**DOI:** 10.1186/s40478-018-0602-5

**Published:** 2018-09-25

**Authors:** Anthony P. Y. Liu, Julie H. Harreld, Lisa M. Jacola, Madelyn Gero, Sahaja Acharya, Yahya Ghazwani, Shengjie Wu, Xiaoyu Li, Paul Klimo, Amar Gajjar, Jason Chiang, Ibrahim Qaddoumi

**Affiliations:** 1 Department of Oncology, St. Jude Children’s Research Hospital, 262 Danny Thomas Place, MS 260, Memphis, 38105-3678 TN USA; 2 Department of Diagnostic Imaging, St. Jude Children’s Research Hospital, Memphis, TN USA; 3 Department of Psychology, St. Jude Children’s Research Hospital, Memphis, TN USA; 4 Department of Radiation Oncology, St. Jude Children’s Research Hospital, Memphis, TN USA; 5 Department of Biostatistics, St. Jude Children’s Research Hospital, Memphis, TN USA; 6 Department of Pathology, St. Jude Children’s Research Hospital, 262 Danny Thomas Place, MS 250, Memphis, 38105-3678 TN USA; 7 Department of Surgery, St. Jude Children’s Research Hospital, Memphis, TN USA; 8 Department of Neurosurgery, University of Tennessee Health Science Center, Memphis, TN USA; 9 Le Bonheur Neuroscience Institute, Le Bonheur Children’s Hospital, Memphis, TN USA; 10 Semmes Murphey Clinic, Memphis, TN USA

**Keywords:** Tectal glioma, Imaging findings, Prognostic factors, Histopathology, DNA methylation profiling, Long-term follow-up

## Abstract

Tectal glioma (TG) is a rare low-grade tumor occurring predominantly in the pediatric population. There has been no detailed analysis of molecular alterations in TG. Risk factors associated with inferior outcome and long-term sequelae of TG have not been well-documented. We retrospectively studied TGs treated or referred for review at St. Jude Children’s Research Hospital (SJCRH) between 1986 and 2013. Longitudinal clinical data were summarized, imaging and pathology specimen centrally reviewed, and tumor material analyzed with targeted molecular testing and genome-wide DNA methylation profiling. Forty-five patients with TG were included. Twenty-six (57.8%) were male. Median age at diagnosis was 9.9 years (range, 0.01–20.5). Median follow-up was 7.6 years (range, 0.5–17.0). The most common presenting symptoms were related to increased intracranial pressure. Of the 22 patients treated at SJCRH, 19 (86%) required cerebrospinal fluid diversion and seven (32%) underwent tumor-directed surgery. Five patients (23%) received radiation therapy and four (18%) systemic therapy. Ten-year overall and progression-free survival were 83.9 ± 10.4% and 48.7 ± 14.2%, respectively. Long-term morbidities included chronic headaches, visual symptoms and neurocognitive impairment. Lesion ≥3cm^2^, contrast enhancement and cystic changes at presentation were risk factors for progression. Among those with tumor tissue available, 83% showed growth patterns similar to pilocytic astrocytoma and 17% aligned best with diffuse astrocytoma. *BRAF* duplication (a marker of *KIAA1549-BRAF* fusion) and *BRAF* V600E mutation were detected in 25% and 7.7%, respectively. No case had histone H3 K27M mutation. DNA methylation profile of TG was distinct from other brain tumors. In summary, TG is an indolent, chronic disease with unique clinical and molecular profiles and associated with long term morbidities. Large size, contrast enhancement and cystic changes are risk factors for progression.

**Electronic supplementary material:**

The online version of this article (10.1186/s40478-018-0602-5) contains supplementary material, which is available to authorized users.

## Introduction

Tectal glioma (TG) is a rare tumor with a predilection for the pediatric population [18]. It involves critical locations in the brainstem including superior and inferior colliculi and the narrow passage of aqueduct of Sylvius. TG may be diagnosed by its typical appearance on imaging and, if biopsied, as a low-grade glioma (LGG) histologically. Given the usual indolent course and risk associated with resection in such an eloquent area, the general recommendation is close observation after CSF diversion for hydrocephalus. Many studies on patient outcome are based on neurosurgical series with possible bias [5, 8, 9, 16, 17, 20, 22, 25, 26, 28, 32, 38, 42, 43, 45, 48]. Data on progression predictors, long-term morbidities, and molecular features of this peculiar group of LGG are lacking. We report comprehensively the clinical, neurocognitive, imaging, histologic and molecular features of TG cases treated or reviewed at St. Jude Children’s Research Hospital (SJCRH) over three decades. We found that while clinically indolent, TG is associated with significant long-term morbidities. Although morphologically similar to pilocytic astrocytoma (PA) of other sites of the central nervous system, TG shows a distinct DNA methylation profile. We have identified large size, contrast enhancement and cystic changes as risk factors for progression.

## Material and methods

### Study population

Forty-five patients with TG treated (*n* = 22) or referred for case review (*n* = 23) at SJCRH between January 1986 and December 2017 were reviewed. Diagnosis was based on typical imaging findings (tumor intrinsic to or centered in the tectal plate) and supported by histopathology when available. Comprehensive clinical, imaging and histopathologic data were reviewed as available. Long-term morbidities, including those affecting neurocognitive function were summarized.

### MRI and image analysis

MR images acquired at diagnosis and first progression, if applicable, were centrally reviewed by a board-certified neuroradiologist (JHH). Each tumor was measured in three orthogonal planes, assessed for T1 and T2 signal intensity and circumscription, and graded for the proportions of cystic and/or enhancing tumor components and enhancement avidity at each time-point. The relative apparent diffusion coefficient (rADC) was calculated relative to normal-appearing cerebellum [23]. Progressive disease (PD) was defined as an increase of ≥25% in the product of the two greatest perpendicular diameters compared to baseline [49].

### Histopathologic and molecular studies

The histopathology of cases with available tissue (*n* = 30) was centrally reviewed by a board-certified neuropathologist (JC). For immunohistochemistry, we used antibodies against GFAP (Ventana, 760–4345, prediluted), Olig2 (Cell Marque, 387 M-15, diluted 1:50), neurofilament (Ventana, 760–2661, prediluted), and Ki67 (Dako, M7240, diluted 1:100). Histone H3 K27M mutant proteins were detected with a rabbit polyclonal antibody (EMD Millipore, ABE419, diluted 1:600). *BRAF* V600E mutant protein was detected with a mouse monoclonal antibody (Ventana, 790–4855, prediluted). Chromosome 7q34 duplication, a surrogate marker for *KIAA1549-BRAF* fusion, was detected by interphase fluorescence in situ hybridization (iFISH) with a probe developed in-house (information available upon request).

### Genome-wide DNA methylation profiling and analysis

Genomic DNA (≥250 ng from each sample) was extracted from formalin-fixed paraffin-embedded (FFPE) tissue from nine TG samples with adequate tissue and analyzed using Illumina Infinium MethylationEPIC BeadChip arrays in accordance with the manufacturer’s instructions. Nineteen non-NF1 hypothalamic PAs (HTPAs, *n* = 9) and cerebellar PAs (CBPAs, *n* = 10) were also retrieved from the institutional tumor bank for comparison. Reference methylation profiles of 8 brain tumor entities (rosette-forming glioneuronal tumor, dysembryoplastic neuroepithelial tumor, ganglioglioma, subependymal giant cell astrocytoma, *MYB*-altered low-grade glioma, histone H3 K27M-mutant diffuse midline glioma, and IDH-mutant diffuse astrocytoma / oligodendroglioma) and normal tissue from the hypothalamus, pons, cerebellum and white matter were obtained from publicly available database for comparison [6]. Array data analysis was performed using R v.3.5.0 with several packages from Bioconductor [36]. Raw signal intensities were obtained from IDAT files by using minfi package v.1.26.0 and normalized by performing background correction and a dye-bias correction for both color channels with the functional normalization method [2, 13]. Poor quality (*P* > 0.01) and failed probes (*n* = 29,567) were removed from the downstream analysis. The following filtering criteria were applied: removal of probes targeting the X and Y chromosomes (*n* = 8971), removal of probes containing single-nucleotide polymorphism (*n* = 13,776), and removal of probes not mapping uniquely to the human reference genome (hg19) allowing for one mismatch (*n* = 3965). In total, 400,253 probes targeting CpG sites were kept. Beta-values of the 1000 most variable CpG sites were derived for further analysis. t-SNE analysis was performed using Rtsne package v.0.13 with theta = 0.0 [24, 44]. Agglomerative nesting hierarchical clustering analysis was performed using cluster package v.2.0.7-1 with Euclidean distances and a generalized average method [27].

### Neuropsychologic evaluation

Cognitive assessments performed at SJCRH were reviewed (*n* = 10) for evidence of long-term neurocognitive impairment. For patients with multiple assessments, data from the most recent assessment were used. The cognitive domains most consistently included for analysis were global intelligence, working memory, processing speed, and academics (i.e., word reading and math calculation). Global intelligence (estimated or full-scale IQ score) was assessed with age-appropriate Wechsler scale or the Differential Abilities Scale, Second Edition [11, 47]. Working memory and processing speed were assessed with age appropriate Wechsler scale. Academics were assessed with Woodcock-Johnson Tests of Achievement, Wide Range Achievement Test, or Wechsler Individual Achievement Test [19, 46, 50]. All age-standardized scores were converted to z-scores (mean = 0 and standard deviation = 1). Impairment was defined as a z-score ≥ -1.33 (ninth percentile).

### Statistical analysis

Statistical analysis was performed with R v.3.5.0. The date of diagnosis was defined as the date of the MRI on which the tumor was first detected. Progressive disease (PD) was defined by radiologic progression, together with clinical deterioration and/or a need for intervention. Tumor size and rADC were compared between baseline and progression by paired t-test. Survival analysis was performed with the Kaplan-Meier method. Overall survival (OS) was determined as the duration between diagnosis and death from any cause or last follow-up, whichever was earlier. Progression-free survival (PFS) was determined as the duration between diagnosis and the first detection of PD, death from any cause, or last follow-up, whichever was earlier. Variables were analyzed for their impact on survival by the log-rank test or the Cox proportional hazards model.

## Results

### Demographic and presenting features

**Fig. 1:**
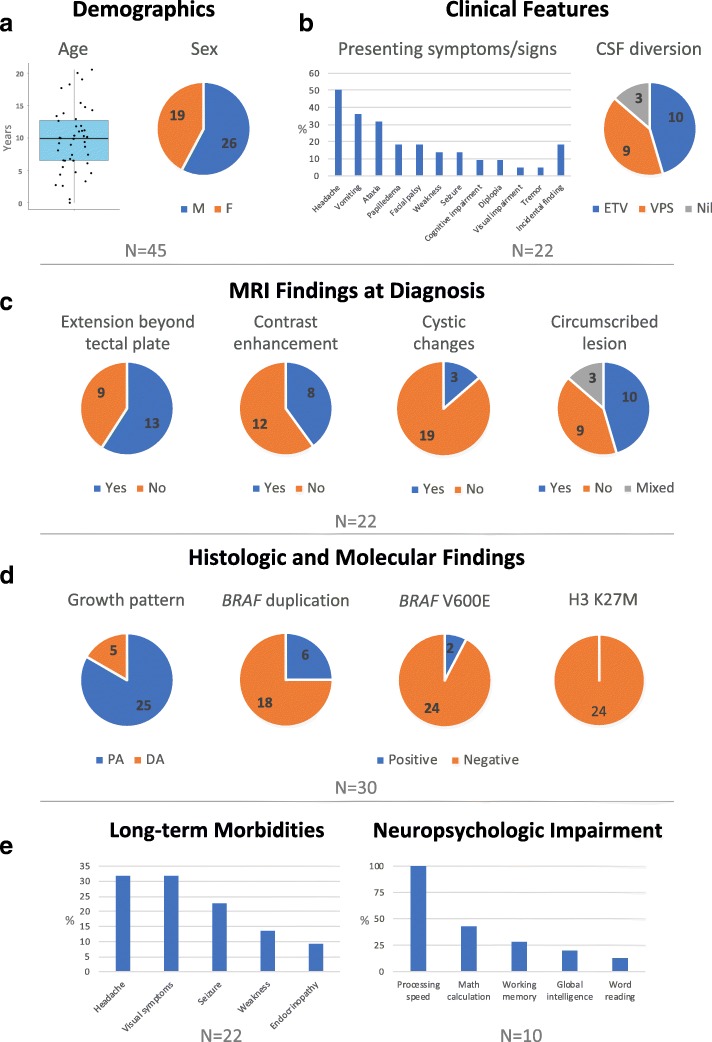
**a** Demographics, **b** clinical features, **c** imaging characteristics, **d** histologic and molecular findings, and **e** long-term morbidities in patients with tectal glioma

Forty-five patients with TG were included in this study (Fig. 1, Additional file 1: Figure S1). Twenty-six (58%) were males, and the median age of diagnosis was 9.9 years (range, 0.01–20.5). Among the 22 patients treated at SJCRH, two had neurofibromatosis type-1 (NF1) and four were diagnosed incidentally: one through antenatal ultrasound and three with failed vision screening (Additional file 2: Table S1). The other patients most commonly presented with headaches (*n* = 11) (Fig. 1). The median duration of symptoms before diagnosis was 0.46 year (range, 1 week-7.3 years). In the three patients for whom presentation preceded diagnosis by ≥6 years, a ventriculoperitoneal (VP) shunt was inserted for hydrocephalus because of a presumed aqueductal stenosis seen on CT; TG was diagnosed only after subsequent MRI (performed because of seizures in two patients and diplopia in one). One out of the 13 patients who underwent complete imaging with MRI brain and spine at diagnosis had evidence of metastasis.

### CSF diversion and surgical and nonsurgical interventions

CSF diversion was required in 19/22 (86%) patients, being performed at presentation in all cases (Fig. 1, Table 1). The initial procedure was an endoscopic third ventriculostomy (ETV) in 10 patients and VP shunting in nine; an Ommaya reservoir was also inserted in eight patients who underwent ETV. Of those who underwent VP shunting, six (67%) required shunt revisions (range, 1–12 times) and one eventually required an ETV. Two patients had a subdural hematoma due to over-shunting, requiring evacuation. Of the patients who had ETV upfront, two (20%) experienced failure necessitating VP shunt placement. Tumor-directed surgery was performed in seven (32%) patients treated at SJCRH. Three had biopsies (two upfront, one at progression), three underwent gross-total resection (GTR) (one upfront, two at progression), and one underwent resection of a spinal metastasis at progression. Two of the patients who underwent GTR developed profound neurologic morbidities due to stroke. Five patients (23%) received focal radiotherapy at 54–55.8Gy: as upfront adjuvant therapy in two cases and at progression in three. One patient suffered from symptomatic radionecrosis requiring steroid and bevacizumab. Systemic therapy was used in four patients (18%) at progression, including one who received RT; a carboplatin-containing regimen was adopted in three out of these patients.

**Table 1:** Interventions and outcomes for patients treated at SJCRH

No.	CSF diversion (no. of revision[s])	Ommaya	Surgery	Chemotherapy	RT	Indication for adjuvant	Long-term sequelae	Outcome	Duration of follow-up (y)
1	VPS (1)	No	Bx upfront	Nil	54 Gy	Upfront	Seizure; memory issues	Alive with SD	12.67
2	VPS (6)	No	Bx at progression, then repeated drainage of cyst; and GTR × 2	POG9060 (ifosfamide)	55.8 Gy	Multiple PD	Learning difficulties	Died of shunt failure	6.78
3	VPS (subdural)	No	Nil	Nil	Nil	N/A	HA + diplopia	Died of obstructive hydrocephalus	7.72
4	VPS (12) → ETV (1)	No	Nil	Carboplatin + tamoxifen	Nil	PD	HA, N, V, nystagmus, OA, VF defect, R CN VII deficit, hypopituitarism	Died of suicide	10.72
5	VPS (subdural)	No	Nil	Nil	Nil	N/A	Nil	Alive with SD	16.98
6	VPS	No	Nil	Nil	Nil	N/A	Spastic quadriplegic CP, epilepsy, severe mental retardation,	Alive with SD	13.97
7	VPS (2)	No	Bx upfront, then resection of spinal metastasis at progression	1. Carboplatin + vincristine + temozolomide 2. Lomustine + procarbazine + vincristine	Nil	PD (metastasis)	GHD, epilepsy off medications	Alive with SD	11.24
8	Nil	No	Nil		Nil	N/A	Migraine	Alive with SD	11.28
9	ETV → VPS at progression	No	Bx upfront, resection at progression (complicated by hemorrhagic stroke)	Nil	54 Gy	Upfront	L hemiparesis, bilateral CN IV and VI palsy	Alive with NED	10.31
10	ETV → VPS (1)	Yes	Bx at progression	1. Carboplatin + vincristine + temozolomide 2. Vinblastine 3. Selumetinib	Nil	PD (metastasis)	HA	Alive with SD on treatment	9.37
11	VPS (2)	No	Bx upfront inconclusive; 2 more Bxs at progression	Nil	54 Gy RT necrosis requiring steroid/bevacizumab	PD	Pathological fracture due to steroid use	Alive with SD	9.57
12	Nil	No	Nil	Nil	54 Gy	PD	Headache, L ptosis, xerostomia, ADHD	Alive with SD	7.56
13	VPS (2)	No	Nil	Nil	Nil	N/A	Seizure, bilateral exotropia, delay, learning difficulty, NF-1 related	Alive with SD	9.81
14	ETV	Yes	Nil	Nil	Nil	N/A	Nil	Alive with SD	5.46
15	ETV	Yes	Nil	Nil	Nil	N/A	Intermittent exotropia bilaterally	Alive with SD	5.81
16	Nil	No	Nil	Nil	Nil	N/A	Migraine	Alive with SD	4.94
17	ETV	Yes	Nil	Nil	Nil	N/A	Memory problem, NF-1 related	Alive with SD	4.51
18	ETV	Yes	Nil	Nil	Nil	N/A	HA? Migraine	Alive with SD	3.64
19	ETV	Yes	Nil	Nil	Nil	N/A	Abnormal EEG	Alive with SD	1.49
20	ETV	Yes	Nil	Nil	Nil	N/A	Nil	Alive with SD	1.49
21	ETV	Yes	Nil	Nil	Nil	N/A	Nil	Alive with SD	1.24
22	ETV	No	Bx upfront	Nil	Nil	N/A	Nil	Alive with SD	0.51

*ADHD* attention-deficit hyperactivity disorder, *Bx* biopsy, *CP* cerebral palsy, *CSF* cerebrospinal fluid, *CN* cranial nerve, *ETV* endoscopic third ventriculostomy, *GHD* growth hormone deficiency, *GTR* gross total resection, *HA* headache, *L* left, *N* nausea, *NF-1* neurofibromatosis type 1, *OA* optic atrophy, *PD* progressive disease, *R* right, *RT* radiotherapy, *SD* stable disease, *V* vomiting, *VF* visual field, *VPS* ventriculo-peritoneal shunt, *y* year(s)

### Disease progression, patient outcome, and long-term morbidities

**Fig. 2:**
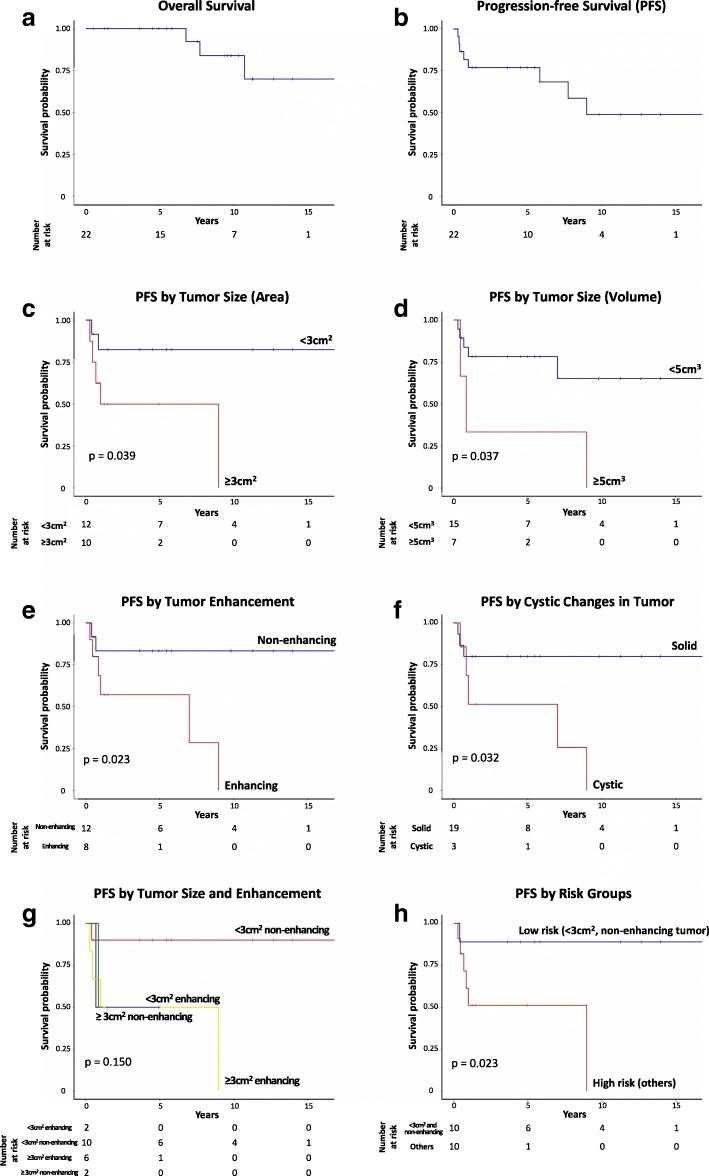
**a** Overall survival and **b** progression-free survival of patients with longitudinal follow-up in our cohort. **c**-**h** Imaging predictors of progression-free survival

During the follow-up period (median, 7.64 years; range, 0.51–16.98), seven patients (32%) treated at SJCRH experienced progression, including two (9%) with metastasis (Table 1). Four patients had a single progression, whereas the remainder experienced two to four progressions. Of the two patients with metastasis, one had known metastatic deposit at the infundibular recess at diagnosis. The second patient did not undergo spinal MRI at diagnosis and was found to have infundibular and spinal metastasis 4 months after diagnosis. The median duration from diagnosis to first progression was 0.68 years (range, 0.28–8.98). Three patients (14%), including two with PD, died (of suicide, obstructive hydrocephalus and suspected shunt failure). This translates into a 5/10-year OS and PFS of 100%/83.9 ± 10.4%, and 76.8 ± 9.1%/48.7 ± 14.2% (Fig. 2). Patients with TG reported significant long-term morbidities, including persistent headaches and visual symptoms (Fig. 1). Cognitive assessments were completed and at a median age of 14.96 years (range, 4.5–24.92) and at a median 5.63 years (range, 0.44–9.55) after diagnosis. Impaired scores were most frequently identified in processing speed (7 out of 7 scores; 100%), working memory (2 out of 7; 28.6%) and academics (math, 3 out of 7, 42.9%). (Fig. 1, Additional file 3: Table S2).

### Imaging features and predictors of progression

**Fig. 3:**
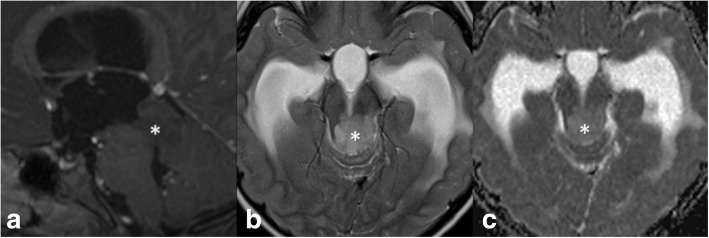
Typical MRI features of tectal glioma. **a** Sagittal post-contrast T1-weighted image shows a typical non-enhancing, T1 hypointense lesion obstructing the cerebral aqueduct (*). **b** Axial T2-weighted image shows typical T2 hyperintensity of the lesion (*), and periventricular CSF accumulation indicative of hydrocephalus. **c** on ADC map, tectal gliomas (*) are typically high in signal (“facilitated” diffusion)

Imaging was available for review in 22 patients (19 patients treated at SJCRH and 3 seen for review). Images obtained at diagnosis revealed that nine patients (40.9%) had lesions confined to the tectal plate, whereas 13 (59.1%) had lesions extending to adjacent structures such as the tegmentum and thalami (Fig. 1, Additional file 4: Table S3). Mean tumor measurements at diagnosis were 4.3(±4.05) cm^2^. All lesions were isointense or hypointense on T1-weighted images and most were hyperintense on T2-weighted sequences (Fig. 3). Contrast enhancement was detected in 8/20 (40%) and cystic changes in 3/22 (14%), the presence of both were significantly correlated with lesions ≥3cm^2^ (*P* = 0.027 and 0.043 respectively). In those who had diffusion-weighted imaging (DWI, *n* = 18), the mean (±SD) relative apparent diffusion coefficient (rADC) was 1.69 (±0.47). Lesions with measured area greater than 3cm^2^ (*P* = 0.023), contrast enhancement (*P* = 0.039), and cystic changes (*P* = 0.037) at diagnosis predicted inferior PFS (Fig. 2). Other radiologic parameters, including tumor extent, tumor circumscription, and rADC values, as well as clinical parameters, namely sex, presenting symptoms, symptom duration before diagnosis and need for CSF diversion, were not significantly associated with PFS. In comparison of sequential MR images at diagnosis and progression (*n* = 8) (Additional file 5: Table S4), mean tumor measurements were 5.48 (±5.54) cm^2^ and 9.61 (±8.35) cm^2^, respectively, with mean increase of 88.19 (±51.49) %. All lesions enhanced at progression, with further increases in the extent and avidity of enhancement in those with pre-existing enhancement. One additional lesion (for a total of four out of eight) developed cystic change at progression, and intra-tumoral hemorrhage was evident in one patient. No significant alteration in rADC was observed at progression (*P* = 0.760).

### Histopathologic and molecular features

**Fig. 4:**
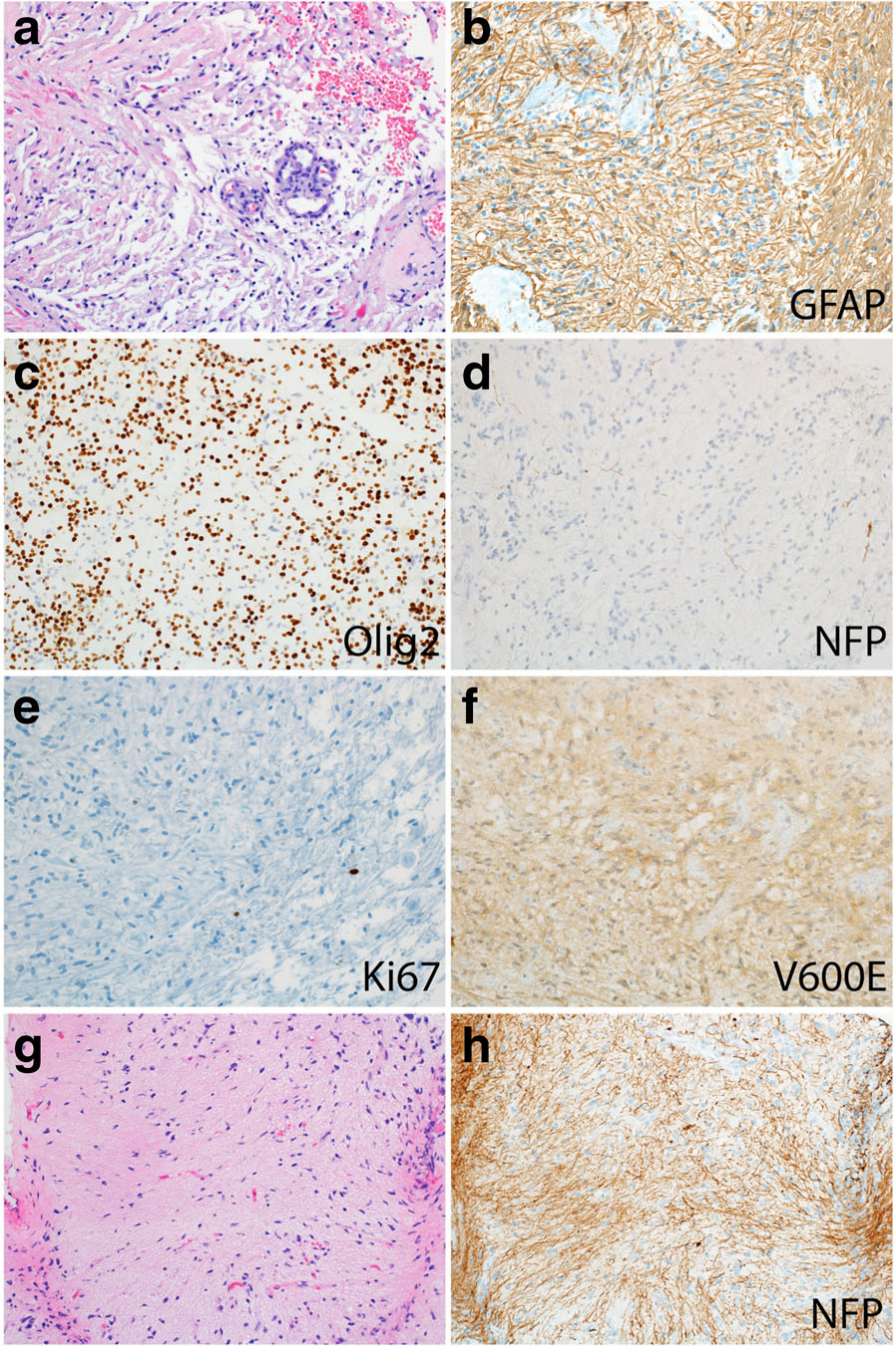
Histologic features of tectal glioma. **a** Most tectal gliomas demonstrate typical morphologic features of pilocytic astrocytoma, including alternating loose and more compact architecture, bland cytology, Rosenthal fibers, and sclerotic vessels, as well as glomeruloid microvascular proliferation. **b**, **c** The tumor cells are diffusely and strongly positive for GFAP and Olig2. **d** Occasional entrapped axons are highlighted by neurofilament (NFP) staining. **e** Ki67 labeling is minimal. **f ***BRAF* V600E mutant protein is detected by immunohistochemical staining in a few cases. **g** Occasionally, tectal glioma may have a more diffuse growth pattern, similar to that of a diffuse astrocytoma, with numerous entrapped axons (highlighted by NFP staining, **h**)

Thirty patients had tumor tissue available for pathology review. They included seven patients treated at SJCRH and 23 referred for case review (Figs 1 and 4, Additional file 6: Table S5). Sixteen patients had tumor samples from initial diagnosis available and 14 had samples obtained at progression. All specimens were classified as LGG and showed bland cytology. Twenty-five samples (83%) displayed histopathological features similar to PA (WHO grade I). These tumors had a non-infiltrative growth pattern with biphasic, alternating loose and more compact architecture, similar to PAs of other sites. The tumor cells had piloid morphology. Microcystic regions, Rosenthal fibers and eosinophilic granular bodies were frequent findings. Five (17%) had histopathological features aligned best with diffuse astrocytoma (DA) (WHO grade II). These tumors had a diffusely infiltrative growth pattern similar to DAs of other sites. Only 1/14 samples obtained at progression (7.1%) exhibited a DA growth pattern, suggesting that such finding does not equate an increased risk of progression. *BRAF* duplication consistent with the presence of *KIAA1549-BRAF* fusion was present in 6/24 samples (25%), whereas *BRAF* V600E mutation was detected in 2/26 samples (7.7%). All eight samples with *BRAF* alterations exhibited a PA-like growth pattern. In contrast, none of the 24 samples evaluated harbored the histone H3 K27M mutation. The prognostic value of *BRAF* alterations could not be meaningfully interpreted with the limited sample size.

### Genome-wide DNA methylation profiling

**Fig. 5:**
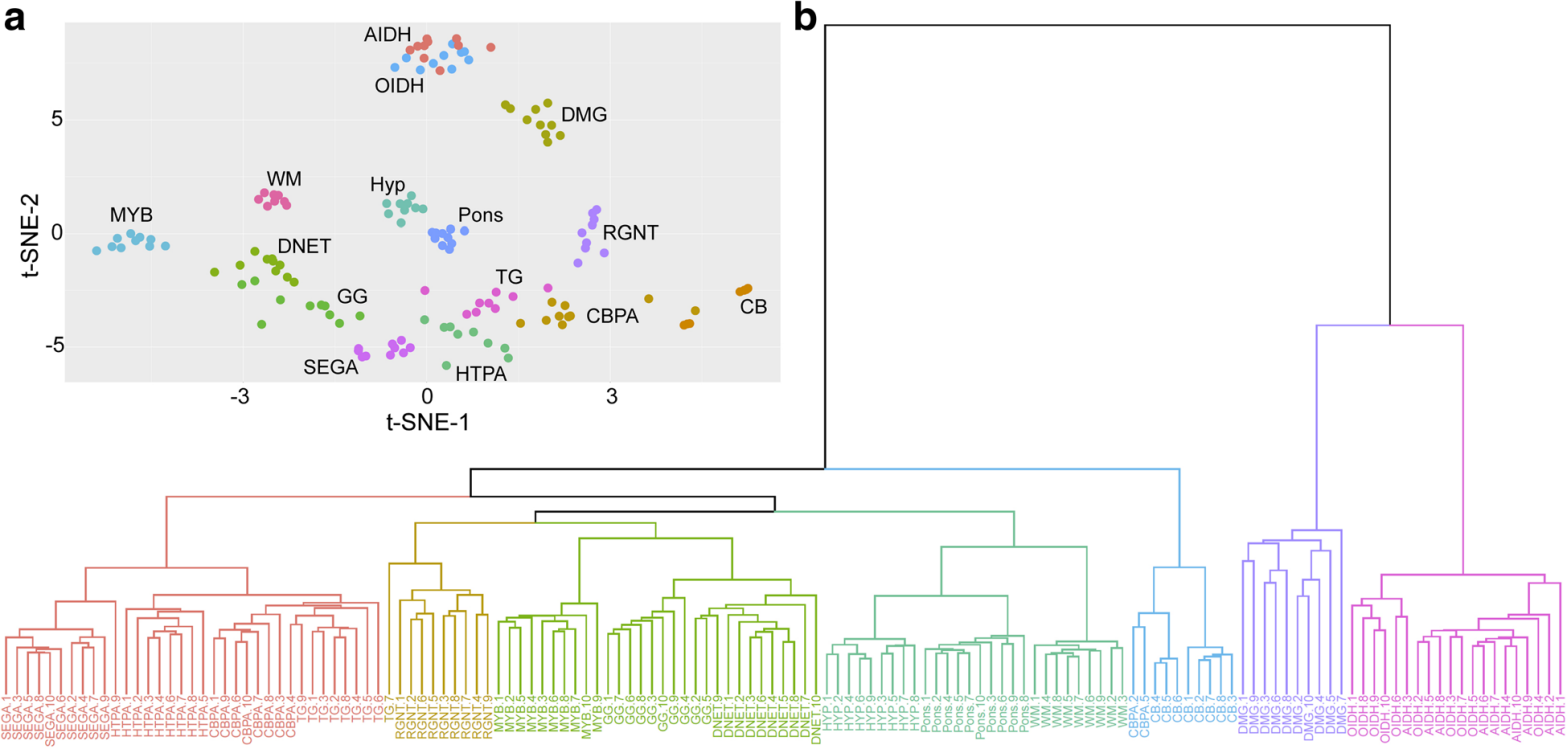
Genome-wide DNA methylation profiling support tectal glioma as a molecularly distinct entity. **a** t-SNE plot and **b** dendrogram of unsupervised cluster analysis comparing DNA methylation profile of tectal glioma with those of 10 other brain tumor entities including PAs of nearby sites (cerebellum, CBPA, and hypothalamus, HTPA), rosette-forming glioneuronal tumor (RGNT), dysembryoplastic neuroepithelial tumor (DNET), ganglioglioma (GG), subependymal giant cell astrocytoma (SEGA), *MYB*-altered LGG, histone H3 K27M-mutant diffuse midline glioma (DMG) and IDH-mutant diffuse astrocytoma (AIDH) / oligodendroglioma (OIDH), and normal tissue from hypothalamus (Hyp), pons, cerebellum (CB) and white matter (WM) demonstrate that tectal glioma forms a distinct cluster

Analysis of genomic DNA methylation profiles by t-SNE plot and unsupervised cluster analysis demonstrated that TG harbors methylation patterns distinct from PAs of nearby sites (cerebellum, CBPA, and hypothalamus, HTPA) and also other brain tumors including rosette-forming glioneuronal tumor (RGNT), dysembryoplastic neuroepithelial tumor (DNET), ganglioglioma (GG), subependymal giant cell astrocytoma (SEGA), *MYB*-altered LGG, histone H3 K27M-mutant diffuse midline glioma (DMG), and IDH-mutant diffuse astrocytoma (AIDH) / oligodendroglioma (OIDH), and normal tissue from the hypothalamus (Hyp), pons, cerebellum (CB) and white matter (WM) (Fig. 5).

## Discussion

We have reported imaging findings, histopathology, molecular analysis and outcomes of children with TG compiled over a period of three decades. The presentation of TG is often typified by symptoms of raised intracranial pressure and delayed diagnosis, with most reports describing a lead time of 3–6 months [1, 4, 5, 8, 15, 16, 37, 39, 41, 43, 45]. Misdiagnosis of TG as aqueductal stenosis based on CT, as in three of our patients, was common and was associated with even longer symptom durations before TG diagnosis [5]. The more widespread application of MRI for neuroimaging has resulted in incidental diagnoses of TG [1, 14, 17, 22, 26, 32–34, 41, 42, 48]. TG was an incidental finding in up to 27% of patients in various series and in an even higher percentage when MRI was part of a structured surveillance, as in children with NF1 [14, 33]. Whether TG diagnosis in this context is truly beneficial for patient outcomes remains uncertain [40].

Initial treatment of pediatric TG presented with obstructive hydrocephalus involves CSF diversion. VPS use has been associated with frequent failures, the need for revision, issues with MR compatibility, and over-shunting [4, 8, 15, 16, 28, 41]. Consequently, ETV has replaced shunt insertion as the preferred method of CSF diversion [9, 20, 26, 48]. Ommaya reservoirs can be safely inserted during ETV, allowing emergent CSF withdrawal in the event of ETV failure [10]. Concomitant tumor resection is best avoided because of its inherent risks [25]. Lapras and colleagues reported their experience in resecting 12 tectal plate lesions upfront, with GTR being achieved in nine patients and partial resection in three [25]. However, this accomplishment was at the expense of a vegetative state and death in one patient and surgical complications requiring early re-operation in four others, as well as other complications including visual-field defects, Parinaud syndrome, and mutism in further patients. Other studies reserved tumor biopsy/resection for disease progression [22, 43]. Similar to our cohort, around one-third of patients in the literature eventually required tumor-directed surgery, and visual deficits, gaze palsies, and intracranial hemorrhages remained significant complications. In view of the significant surgical morbidities, biopsy or resection of TG should only be reserved for tumors with an atypical radiographic appearance, and for debulking as well as to guide targeted treatment (such as *BRAF* and *MEK* inhibitors) at progression [5, 39, 42].

Adjuvant therapy with chemotherapy and/or focal radiation is often employed in patients with PD [4, 5, 14, 17, 21, 25, 30, 32, 34, 38, 41–43, 45]. In our study, significant predictors of progression included tumor size greater than 3cm^2^, contrast enhancement and cystic changes at diagnosis, confirming the suggestions of previous reports [22, 34, 43]. To evaluate the role of adjuvant therapy and treatment outcome, we extensive reviewed clinical reports on pediatric TG (< 21 years at diagnosis, 5 or more patients) and combined with data from our cohort (Additional file 7: Table S6) [1, 4, 5, 7–9, 14–17, 20–22, 25, 26, 28, 30, 32–34, 37–39, 41–43, 45, 48]. Among 26 studies reporting details of adjuvant therapy, 56/463 patients (12.1%) received focal radiation with doses of 50.2–56.8Gy, whereas 26/463 (5.6%) received systemic therapy [1, 4, 5, 8, 9, 14–17, 21, 25, 26, 28, 30, 32–34, 37–39, 41–43, 45, 48]. Patient outcomes were reported in 28 studies, with 495/508 patients (97.4%) surviving for average durations ranging from 2 to 10 years at follow-up [1, 4, 5, 8, 9, 14–17, 20–22, 25, 26, 28, 30, 32–34, 37–39, 41–43, 45, 48]. In the studies describing PD (*n* = 24), 121 of 453 patients (26.7%) displayed clinical and/or radiographic PD, with the average duration from diagnosis to progression ranging from 3 months to 7.8 years [1, 4, 5, 8, 9, 14–17, 20–22, 28, 30, 32–34, 38, 39, 41–43, 48]. Of the 13 patients who died, eight died of PD (one had high-grade glioma [HGG]), one died of metastatic neuroblastoma, one died of VPS infection, and three deaths were from our series discussed earlier. These data suggest that the vast majority of children with TG, despite the risk of progression in a quarter, are long-term survivors with salvage adjuvant treatment. Such findings are striking in the context of most TG not being surgically removed and the extent of resection being one of the most important prognostic factors in other LGGs [35].

The unique clinical behavior of TG might be explained by its anatomical location and the differences in tumor biology among LGGs from various body sites. In our study, we for the first time interrogated the molecular distinctiveness of TG by performing targeted studies (of *BRAF* alterations and histone mutations) and genome-wide DNA methylation profiling. The frequency of *KIAA1549-BRAF* fusion (by the presence of *BRAF* locus duplication on iFISH) in TG (25%) appeared to be lower than that in PAs from the cerebellum (92%) and supratentorium (59%), whereas the frequency of *BRAF* V600E mutation (7.7%) appeared to be intermediate between the two (0% and 10% respectively) [3, 52]. Despite the extra-tectal extension of a proportion of tumors, the lack of histone H3 K27M mutations in all 24 samples supported the biological distinctiveness of TG from other midline diffuse gliomas of the brainstem, which are often characterized by such histone mutations and a more aggressive clinical course. DNA methylation profiling has established a role in defining clinically relevant subgroups in CNS tumors such as medulloblastoma, ependymoma and HGG [12, 31, 51]. Our comparison of the methylation profiles of TG and cerebellar/hypothalamic PAs revealed molecular heterogeneity among these morphologically similar lesions, further supporting the biological uniqueness of TG.

Pediatric TG should be considered a chronic disease, in which care for long-term morbidities is of paramount importance. Caregivers should be informed of the common long term morbidities in patients with TG including chronic headache, persistent visual symptoms and neurocognitive impairments [1, 4, 14, 16, 20, 22, 25, 28, 29, 39, 43, 48]. Neuropsychologic assessments in our cohort suggested areas of deficit in working memory, processing speed and academics, specifically math, thus adding to prior reports of problems in visual attention deficits, behavior problems, and academic achievement, calling for neuropsychologic evaluation as standard of care in patients with TG [1, 14]. Despite the retrospective nature of our analysis and limitation on available material and follow-up information on some of the cases, we comprehensively addressed the clinical, imaging, histologic and molecular distinctiveness of TG. Our findings provide evidence supporting TG as a distinct diagnostic entity.

## Conclusion

Tectal glioma is a clinically indolent disease and biologically distinct from other LGGs. Symptoms are frequently due to obstructive hydrocephalus and diagnosis can be made based on typical MRI features. CSF diversion by ETV is sufficient for most patients. Disease progression may be predicted by size, contrast enhancement and cystic change on initial MRI. Long-term follow-up for morbidities including neuropsychologic impairments is necessary for patients with this chronic illness.

## Additional files

Additional file 1: **Figure S1.** Number of patients who underwent clinical, radiologic and pathologic review in our cohort. (TIF 881 kb)

Additional file 2: **Table S1.** Demographics and presenting symptoms in patients treated at SJCRH. (DOCX 23 kb)

Additional file 3: **Table S2.** Characteristics of patients who underwent neuropsychologic testing in our cohort. (DOCX 22 kb)

Additional file 4: **Table S3.** Centrally reviewed diagnostic imaging features in our study cohort. (DOCX 27 kb)

Additional file 5: **Table S4.** Evolution of imaging features at disease progression. (DOCX 24 kb)

Additional file 6: **Table S5.** Histopathologic features and molecular findings. (DOCX 24 kb)

Additional file 7: **Table S6.** Summary of literature on pediatric tectal glioma. (DOCX 47 kb)
