# Supplementary figures and images for: Tectal glioma as a distinct diagnostic entity: a comprehensive clinical, imaging, histologic and molecular analysis
Source: Acta Neuropathol Commun. 2018 Sep 25;6:101. doi: 10.1186/s40478-018-0602-5 (PMC6154813; doi:10.1186/s40478-018-0602-5)

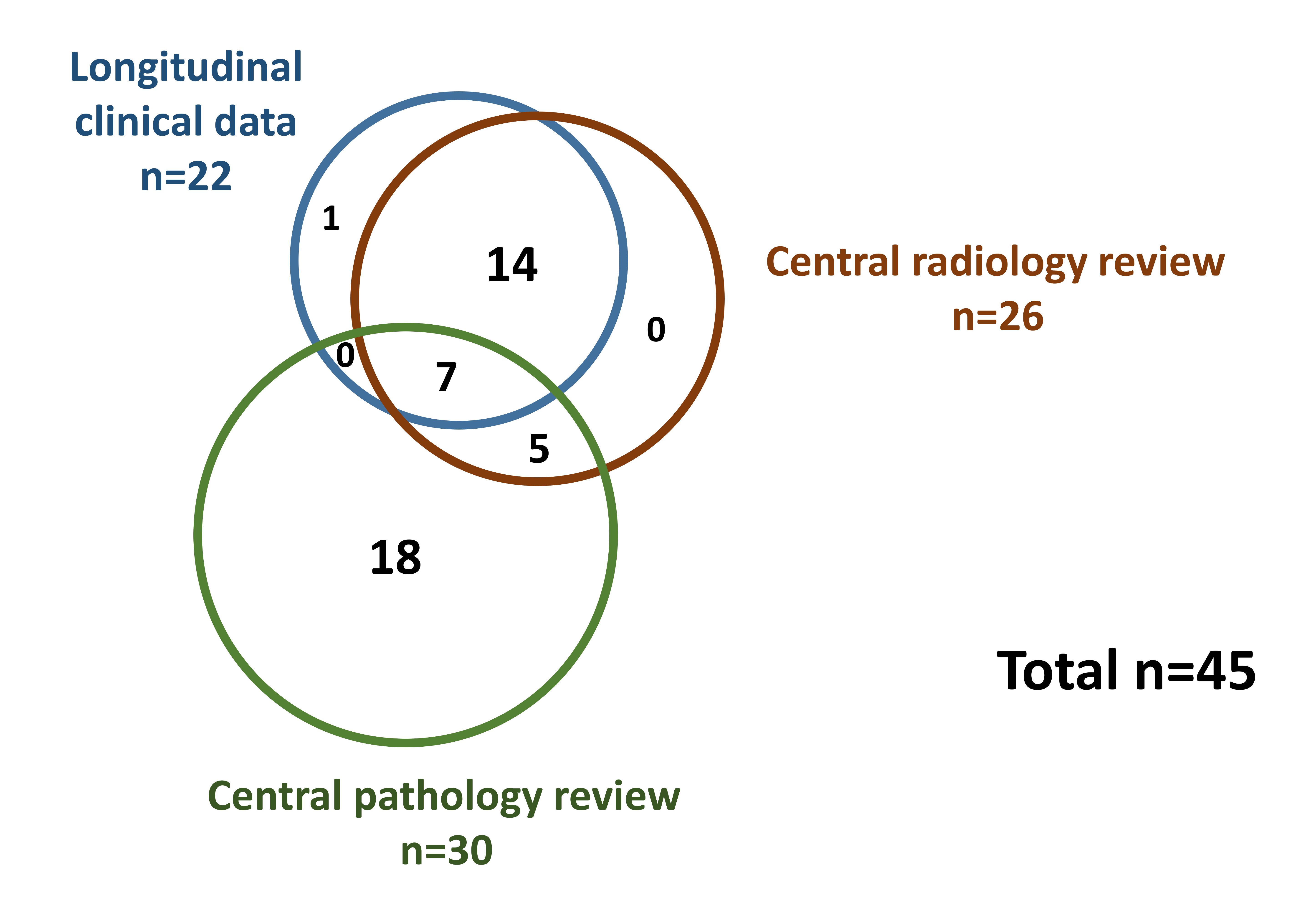

Supplement: Supplementary file 1 — Figure S1. Number of patients who underwent clinical, radiologic and pathologic review in our cohort. (TIF 881 kb) [file 40478_2018_602_MOESM1_ESM.tif]
