# Supplementary material for: Tectal glioma as a distinct diagnostic entity: a comprehensive clinical, imaging, histologic and molecular analysis
Source: Acta Neuropathol Commun. 2018 Sep 25;6:101. doi: 10.1186/s40478-018-0602-5 (PMC6154813; doi:10.1186/s40478-018-0602-5)
Supplement: Supplementary file 2 — Table S1. Demographics and presenting symptoms in patients treated at SJCRH. (DOCX 23 kb) [file 40478_2018_602_MOESM2_ESM.docx]

| No. | Age at Dx (y) | Sex | Presenting symptoms | | | | | | | | | | | | Duration of symptom before Dx (y) | NF1 |
| --- | --- | --- | --- | --- | --- | --- | --- | --- | --- | --- | --- | --- | --- | --- | --- | --- |
|  |  |  | **HA** | **Vomiting** | **Visual disturbance** | **Diplopia** | **Papilledema** | **Ataxia** | **Tremor** | **Seizure** | **Cognitive** | **Weakness** | **Facial palsy** | **Incidental** |  |  |
| 1 | 13.77 | M | Y | Y | N | N | N | N | N | Y | N | N | N | N | 6 | No |
| 2 | 12.88 | M | Y | Y | N | Y | N | N | N | Y | N | N | Y | N | 7.3 | No |
| 3 | 19.00 | M | Y | Y | N | Y | N | Y | N | N | Y | N | N | N | 7 | No |
| 4 | 6.77 | F | Y | Y | N | N | N | N | N | N | N | N | N | N | 0.46 | No |
| 5 | 9.19 | M | N | N | N | N | Y | Y | N | N | Y | Y | Y | N | 0.5 | No |
| 6 | 10.96 | M | N | N | N | N | N | N | N | Y | N | N | N | N | 0.25 | No |
| 7 | 2.74 | M | Y | N | N | N | N | Y | N | N | N | N | N | N | 0.17 | No |
| 8 | 6.55 | M | Y | N | N | N | N | N | N | N | N | N | N | N | 4 | No |
| 9 | 15.37 | M | N | N | Y | N | Y | N | N | N | N | N | N | Y | N/A | No |
| 10 | 6.09 | M | Y | N | N | N | N | Y | N | N | N | N | N | N | 0.02 | No |
| 11 | 8.65 | F | Y | Y | N | N | N | N | N | N | N | N | N | N | 0.46 | No |
| 12 | 9.97 | M | N | Y | N | N | N | N | N | N | N | N | N | N | 3 | No |
| 13 | 0.01 | F | N | N | N | N | N | N | N | N | N | N | N | Y | N/A | Yes |
| 14 | 9.38 | M | N | N | N | N | N | Y | Y | N | N | N | N | N | 0.08 | No |
| 15 | 11.83 | M | Y | Y | N | N | N | N | N | N | N | N | N | N | 0.17 | No |
| 16 | 19.99 | F | Y | Y | N | N | N | N | N | N | N | N | N | N | 0.2 | No |
| 17 | 4.35 | F | N | N | Y | N | Y | N | N | N | N | N | N | Y | N/A | Yes |
| 18 | 11.76 | M | Y | N | N | N | N | N | N | N | N | N | Y | N | 1.5 | No |
| 19 | 3.31 | F | N | N | N | N | N | N | N | N | N | Y | Y | N | 0.25 | No |
| 20 | 18.22 | F | N | N | N | N | Y | N | N | N | N | N | N | Y | N/A | No |
| 21 | 14.73 | M | N | N | N | N | N | Y | N | N | N | N | N | N | 1 | No |
| 22 | 5.52 | M | N | N | Y | N | N | Y | N | N | N | Y | N | N | 0.33 | No |

**Table S1. Demographics and presenting symptoms in patients treated at SJCRH**

Dx, diagnosis; F, female; HA, headache; M, male; N, no; NF1, neurofibromatosis type 1; No., number; Y, yes; y, year(s)
