# Supplementary material for: Tectal glioma as a distinct diagnostic entity: a comprehensive clinical, imaging, histologic and molecular analysis
Source: Acta Neuropathol Commun. 2018 Sep 25;6:101. doi: 10.1186/s40478-018-0602-5 (PMC6154813; doi:10.1186/s40478-018-0602-5)
Supplement: Supplementary file 3 — Table S2. Characteristics of patients who underwent neuropsychologic testing in our cohort. (DOCX 22 kb) [file 40478_2018_602_MOESM3_ESM.docx]

**Table S2.** Neuropsychological testing in patients with tectal glioma illustrating impairment in a significant proportion – (A) characteristics of tested patients and (B) summary of results

**(A)**

| **Sex** | **Age at diagnosis (y)** | | | **Age at assessment (y)** | | | | **Interval from diagnosis to assessment (y)** | | |  |
| --- | --- | --- | --- | --- | --- | --- | --- | --- | --- | --- | --- |
| M | 15.37 | | | 24.92 | | | | 9.55 | | |  |
| M | 13.77 | | | 16.83 | | | | 3.06 | | |  |
| F | 4.35 | | | 8.33 | | | | 3.98 | | |  |
| F | 0.01 | | | 7.33 | | | | 7.32 | | |  |
| M | 9.97 | | | 17.25 | | | | 7.28 | | |  |
| F | 8.65 | | | 18.17 | | | | 9.52 | | |  |
| M | 6.09 | | | 14.75 | | | | 8.66 | | |  |
| M | 9.38 | | | 12.17 | | | | 2.79 | | |  |
| F | 3.31 | | | 4.50 | | | | 1.19 | | |  |
| M | 14.73 | | | 15.17 | | | | 0.44 | | |  |
| M, male; F, female; y, years | | | | | | | | | | | |
| **(B)** | |  | | | |  |  |  |  |  |  |
|  | | | **n** | | **%** | | **Mean** | | **SD** | **% Impaired^a^** | |
| Global intelligence | | | 10 | | 100 | | -0.92 | | 0.83 | 20.0 | |
| Working memory | | | 7 | | 70 | | -0.71 | | 1.01 | 28.6 | |
| Processing speed | | | 7 | | 70 | | -1.90 | | 0.57 | 100.0 | |
| Academics | | |  | |  | |  | |  |  | |
| Word reading | | | 8 | | 80 | | -0.23 | | 1.18 | 12.5 | |
| Math calculation | | | 7 | | 70 | | -0.57 | | 1.74 | 42.9 | |

SD = standard deviation. Mean, SD, and mean difference are reported in z-scores, with a normative mean of 1 and a standard deviation of 0.5. ^a^ Frequency of z-score ≤ −1.33, tenth percentile).
