# Supplementary material for: Tectal glioma as a distinct diagnostic entity: a comprehensive clinical, imaging, histologic and molecular analysis
Source: Acta Neuropathol Commun. 2018 Sep 25;6:101. doi: 10.1186/s40478-018-0602-5 (PMC6154813; doi:10.1186/s40478-018-0602-5)
Supplement: Supplementary file 4 — Table S3. Centrally reviewed diagnostic imaging features in our study cohort. (DOCX 27 kb) [file 40478_2018_602_MOESM4_ESM.docx]

| **Extent of lesion** | **Measurement 1 (cm)** | **Measurement 2 (cm)** | | **Measurement 3 (cm)** | | **2D measurement (cm2)** | | **T1** | | **T2** | **Enhancement (proportion)*** | **Enhancement (avidity)#** | **Circumscribed lesion** | **rADC** | **Cystic changes*** | **PD** |
| --- | --- | --- | --- | --- | --- | --- | --- | --- | --- | --- | --- | --- | --- | --- | --- | --- |
| tectum | 1.8 | | 0.9 | | 0.9 | | 1.62 | | iso | hyper | 0 | 0 | Y | N/A | 0 | N |
| tectum, thalamus | 1.7 | | 1.4 | | 1.7 | | 2.89 | | iso/hypo | hyper | 0 | 0 | N | N/A | 0 | N |
| tectum, thalamus | 1.6 | | 0.9 | | 0.8 | | 1.44 | | iso/hypo | hyper | 0 | 0 | Y | N/A | 0 | N |
| tectum, tegmentum, thalamus | 1.4 | | 0.9 | | 1.4 | | 1.96 | | iso/hypo | hyper | 0 | 0 | N | 1.54 | 0 | N |
| tectum | 1.6 | | 0.9 | | 1.5 | | 2.4 | | iso | iso | N/A | N/A | Y | 0.91 | 0 | N |
| tectum | 2 | | 0.9 | | 1.4 | | 2.8 | | iso | mixed | 0 | 0 | N | 1.47 | 0 | N |
| tectum | 1.7 | | 1.1 | | 1.5 | | 2.55 | | iso | hyper | 0 | 0 | N | 1.43 | 0 | N |
| tectum | 1.7 | | 1 | | 1.4 | | 2.38 | | hypo | hyper | 1 | 2 | Y | 1.70 | 0 | N |
| tectum | 1.2 | | 0.5 | | 0.7 | | 0.84 | | hypo | hyper | 0 | 0 | N | 1.58 | 0 | N |
| tectum | 1.8 | | 1.1 | | 1.4 | | 2.52 | | iso | hyper | 0 | 0 | Y | 1.51 | 0 | N |
| tectum, tegmentum, thalamus | 3.6 | | 2.2 | | 3.7 | | 13.32 | | hypo | hyper | 0 | 0 | Y | 1.56 | 0 | N |
| tectum, dorsal tegmentum | 2.9 | | 1.4 | | 1.3 | | 4.06 | | iso | iso | 1 | 2 | N | 1.32 | 0 | N |
| tectum, periaqueductal, thalamus | 1.7 | | 1.8 | | 1 | | 3.06 | | hypo | hyper | 0 | 0 | Mixed | 2.90 | 0 | N |
| tectum, tegmentum | 1.7 | | 1.5 | | 1.9 | | 3.23 | | hypo | hyper | 1 | 3 | N | 1.46 | 0 | N |
| tectum, tegmentum | 1.5 | | 1.4 | | 2.1 | | 3.15 | | hypo | hyper | 2 | 2 | Y | 1.90 | 0 | Y |
| tectum, vermis | 2.8 | | 3.1 | | 2.5 | | 8.68 | | hypo | hyper | 2 | 2 | N | N/A | 2 | Y |
| tectum, tegmentum, thalamus | 5.6 | | 3.2 | | 3.1 | | 17.92 | | hypo | mixed | 3 | 2 | Y | 1.61 | 3 | Y |
| tectum | 0.7 | | 0.7 | | 0.7 | | 0.49 | | hypo | hyper | 4 | 4 | Y | 2.40 | 0 | Y |
| tectum | 1.8 | | 1.2 | | 1.2 | | 2.16 | | hypo | hyper | 0 | 0 | Y | 2.09 | 0 | Y |
| tectum, thalamus | 2.2 | | 1.7 | | 1.7 | | 3.74 | | iso | hyper | 2 | 3 | Mixed | 1.34 | 0 | Y |
| tectum, thalamus | 1.6 | | 1.4 | | 2.3 | | 3.68 | | hypo | hyper | 0 | 0 | Mixed | 2.50 | 3 | Y |
| tectum, thalamus, vermis | 2.1 | | 1.9 | | 1.8 | | 3.99 | | iso | hyper | N/A | N/A | N | 1.37 | 0 | Y |

**Table S3: Centrally reviewed diagnostic radiographic features in our study cohort**

*Graded for proportions of cystic and/or enhancing tumor components: 1=<25%, 2=25-49%, 3=50-75%, 4=>75%

#Grading for avidity of enhancement: 0=none, 1=minimal, 2=mild, 3=moderate, 4=significant/bright

2D, two dimensional; hyper, hyperintense; hypo, hypointense; iso, isointense; N, no; N/A, not available; PD, progressive disease; rADC, relative apparent diffusion coefficient; Y, yes
