# Supplementary material for: Tectal glioma as a distinct diagnostic entity: a comprehensive clinical, imaging, histologic and molecular analysis
Source: Acta Neuropathol Commun. 2018 Sep 25;6:101. doi: 10.1186/s40478-018-0602-5 (PMC6154813; doi:10.1186/s40478-018-0602-5)
Supplement: Supplementary file 5 — Table S4. Evolution of imaging features at disease progression. (DOCX 24 kb) [file 40478_2018_602_MOESM5_ESM.docx]

|  |  |  |  |  |  | **At diagnosis** | |  |  |  |  |  |  |  |  |  |  | **At progression** | |  |  |  |  |
| --- | --- | --- | --- | --- | --- | --- | --- | --- | --- | --- | --- | --- | --- | --- | --- | --- | --- | --- | --- | --- | --- | --- | --- |
| **Extent of lesion** | **Measurement 1 (cm)** | **Measurement 2 (cm)** | **Measurement 3 (cm)** | **2D measurement (cm2)** | **T1** | **T2** | **Enhancement (proportion)*** | **Enhancement (avidity)#** | **Circumscription** | **rADC** | **Cystic changes*** | **Extent of lesion** | **Measurement 1 (cm)** | **Measurement 2 (cm)** | **Measurement 3 (cm)** | **2D measurement (cm2)** | **T1** | **T2** | **Enhancement (proportion)*** | **Enhancement (avidity)#** | **Circumscription** | **rADC** | **Cystic changes*** |
| tectum, tegmentum | 1.5 | 1.4 | 2.1 | 3.15 | hypo | hyper | 2 | 2 | Y | 1.90 | 0 | tegmentum | 2.1 | 2 | 1.5 | 4.2 | Hypo | Hyper | 4 | 3 | Y | 1.79 | 0 |
| tectum, vermis | 2.8 | 3.1 | 2.5 | 8.68 | hypo | hyper | 2 | 2 | N | N/A | 2 | tectum, vermis | 3.9 | 3.5 | 3.4 | 13.65 | Mixed | Mixed | 4 | 3 | Y | N/A | 4 |
| tectum, tegmentum, thalamus | 5.6 | 3.2 | 3.1 | 17.92 | hypo | mixed | 3 | 2 | Y | 1.61 | 3 | tectum, tegmentum, thalamus, pons, MCP | 5.5 | 3.8 | 5 | 27.5 | Hypo | Hyper | 2 | 2 | N | 1.42 | 1 |
| tectum | 0.7 | 0.7 | 0.7 | 0.49 | hypo | hyper | 4 | 4 | Y | 2.45 | 0 | tectum | 1 | 1 | 1 | 1 | Hypo | Hyper | 4 | 4 | Y | N/A |  |
| tectum | 1.8 | 1.2 | 1.2 | 2.16 | hypo | hyper | 0 | 0 | Y | 2.09 | 0 | tectum | 2.1 | 1.2 | 1.3 | 2.73 | Hypo | Hyper | 1 | 2 | Y | 1.58 | 0 |
| tectum, thalamus | 2.2 | 1.7 | 1.7 | 3.74 | iso | hyper | 2 | 3 | Mixed | 1.34 | 0 | tectum, thalami | 2.7 | 3.3 | 2.3 | 8.91 | Hypo | Mixed | 3 | 4 | Mixed | 1.54 | 0 |
| tectum, thalamus | 1.6 | 1.4 | 2.3 | 3.68 | hypo | hyper | 0 | 0 | Mixed | 2.50 | 3 | tectum, thalamus, peduncle | 2.7 | 2 | 3.3 | 8.91 | Hypo | Hyper | 3 | 3 | N | 3.41 | 4 |
| tectum, thalamus, vermis | 2.1 | 1.9 | 1.8 | 3.99 | iso | hyper | N/A | N/A | N | 1.37 | 0 | tectum, tegmentum, vermis, thalamus | 4 | 2.4 | 2.5 | 10 | Mixed | Mixed | 2 | 2 | Y | 1.45 | 2 |

**Table S4. Evolution of radiological features at disease progression**

*Graded for proportions of cystic and/or enhancing tumor components: 1=<25%, 2=25-49%, 3=50-75%, 4=>75%

#Grading for avidity of enhancement: 0=none, 1=minimal, 2=mild, 3=moderate, 4=significant/bright
