# Supplementary material for: Tectal glioma as a distinct diagnostic entity: a comprehensive clinical, imaging, histologic and molecular analysis
Source: Acta Neuropathol Commun. 2018 Sep 25;6:101. doi: 10.1186/s40478-018-0602-5 (PMC6154813; doi:10.1186/s40478-018-0602-5)
Supplement: Supplementary file 6 — Table S5. Histopathologic features and molecular findings. (DOCX 24 kb) [file 40478_2018_602_MOESM6_ESM.docx]

**Table S5: Histopathological features and molecular findings of TG**

| **Classification** | | **WHO Grade** | | ***BRAF* duplication** | | ***BRAF* V600E** | | **H3K27M** | | **Growth Pattern** | | **Rosenthal Fibers** | | **EGBs** | | **Sclerotic / Glomeruloid Vessels** | |
| --- | --- | --- | --- | --- | --- | --- | --- | --- | --- | --- | --- | --- | --- | --- | --- | --- | --- |
| **PA** | **DA** | **I** | **II** | **+** | **-** | **+** | **-** | **+** | **-** | **Biphasic** | **Infiltrative** | **+** | **-** | **+** | **-** | **+** | **-** |
|  | |  | |  | |  | |  | |  | |  | |  | |  | |
|  | |  | |  | |  | |  | |  | |  | |  | |  | |
|  | |  | |  | |  | |  | |  | |  | |  | |  | |
|  | |  | |  | |  | |  | |  | |  | |  | |  | |
|  | |  | |  | |  | |  | |  | |  | |  | |  | |
|  | |  | |  | |  | |  | |  | |  | |  | |  | |
|  | |  | |  | |  | | n/d | |  | |  | |  | |  | |
|  | |  | |  | |  | | n/d | |  | |  | |  | |  | |
|  | |  | |  | |  | |  | |  | |  | |  | |  | |
|  | |  | |  | |  | |  | |  | |  | |  | |  | |
|  | |  | |  | |  | |  | |  | |  | |  | |  | |
|  | |  | |  | |  | |  | |  | |  | |  | |  | |
|  | |  | |  | |  | |  | |  | |  | |  | |  | |
|  | |  | |  | |  | |  | |  | |  | |  | |  | |
|  | |  | |  | |  | |  | |  | |  | |  | |  | |
|  | |  | |  | |  | |  | |  | |  | |  | |  | |
|  | |  | |  | |  | |  | |  | |  | |  | |  | |
|  | |  | |  | |  | |  | |  | |  | |  | |  | |
|  | |  | |  | |  | |  | |  | |  | |  | |  | |
|  | |  | |  | |  | |  | |  | |  | |  | |  | |
|  | |  | | Insufficient | |  | |  | |  | |  | |  | |  | |
|  | |  | | Insufficient | | n/d | | n/d | |  | |  | |  | |  | |
|  | |  | | n/d | | n/d | | n/d | |  | |  | |  | |  | |
|  | |  | | n/d | | n/d | | n/d | |  | |  | |  | |  | |
|  | |  | | n/d | | n/d | | n/d | |  | |  | |  | |  | |
|  | |  | |  | |  | |  | |  | |  | |  | |  | |
|  | |  | |  | |  | |  | |  | |  | |  | |  | |
|  | |  | |  | |  | |  | |  | |  | |  | |  | |
|  | |  | |  | |  | |  | |  | |  | |  | |  | |
|  | |  | | n/d | |  | |  | |  | |  | |  | |  | |

DA, diffuse astrocytoma; EGBs, eosinophilic granular bodies; n/d, not done; PA, pilocytic astrocytoma
