# Supplementary material for: Tectal glioma as a distinct diagnostic entity: a comprehensive clinical, imaging, histologic and molecular analysis
Source: Acta Neuropathol Commun. 2018 Sep 25;6:101. doi: 10.1186/s40478-018-0602-5 (PMC6154813; doi:10.1186/s40478-018-0602-5)
Supplement: Supplementary file 7 — Table S6. Summary of literature on pediatric tectal glioma. (DOCX 47 kb) [file 40478_2018_602_MOESM7_ESM.docx]

**Table S6. Summary of literature on pediatric tectal glioma**

| Authors | Year | N | Median age in years at diagnosis (range) | Male | | Pathology | | CSF diversion | Biopsy/resection | RT | | Chemotherapy | Progressive  disease | | | Survival | | | Median follow-up (range) |  |
| --- | --- | --- | --- | --- | --- | --- | --- | --- | --- | --- | --- | --- | --- | --- | --- | --- | --- | --- | --- | --- |
| Current study | 2018 | 22 | 9.68  (0.01–19.99) | | 15 (68%) | | PA = 7 | 19 (86%)  VPS = 9; 1 converted to ETV  ETV = 10; 2 converted to VPS | 7 (32%)  Bx = 3  GTR = 3  Partial resection (spinal metastasis) = 1 | | 5 (23%) | 4 (18%) | | | 7 (32%)  Clinical  Median time to progression = 0.68 y (0.28–8.98) | 19/22 (86%)  1 died of suicide  1 died of shunt failure  1 died of obstructive hydrocephalus | | 7.64 y  (0.51–16.98) | | |
| Kaufmann et al. ^21^ | 2018 | 71 | 9.7 (0.1-17.5) | | 42 (59%) | | PA = 20  DA = 8  Ganglioglioma = 2  RGNT = 1  LGG, NOS = 3  Inadequate = 5 | 63 (89%)  ETV = 29 (41%)  VPS = 14 (20%)  > 1 procedure = 20 (28%) | 39 (55%)  At diagnosis:  Bx = 12  Resection = 15 | | 11 (15%) | 4 (6%) | | | 27/71 (38%)  10 y EFS = 40% | 69/71 (97%)  1 died of PD  1 died of VPS infection | | 6.3 y for surviving patients | | |
| Kershenovich et al.^22^ | 2016 | 39 | 9  (0.3–18) | | 22 (55%) | | PA = 10 LGG = 1 CPC = 1 Astroblastoma = 1 Inadequate = 1 | 35 (88%)  26 with initial ETV; 6 required conversion to VPS 9 with initial VPS; 2 required conversion to ETV | 14 (35%)  Resection = 10 Bx = 2 Bx then 2nd resection = 2 | | Adjuvant in 6 patients (15%)—no details | Adjuvant in 6 patients (15%)—no details | | | 10/19 (52.6%) Radiologic Median time to progression = 56.5 mos | 39/39 (100%)  (long-term data in 39) | | 4 y (1–16)  (n = 23) | | |
| Gass et al.^14^ | 2015 | 26 | 10  (1–17) | | 12 (46%) | | Fibrillary astrocytoma = 1 PA with focal features of pilomyxoid astrocytoma = 1 PA = 1 | 19 (73%)  17 with initial ETV; 3 required conversion to VPS 2 with initial EVD; then converted to ETV/VPS | Bx = 3 | | 4 (15%)  (50.4 Gy);  1 also had SRS | 4 (15%)  Carboplatin + vincristine = 3 Carboplatin → temozolomide → Bevacizumab + irinotecan = 1 | | | 6/26 (23%) Clinical Medium time to progression = 42 mos (5–53) | 25/26 (96%) 1 (4%) died of metastatic neuroblastoma | | 3.8 y  (0.7–11.9) | | |
| Mottolese et al.^30^ | 2015 | 27 | 7.3 (mean) | | 12 (44%) | | PA = 56% Astrocytoma grade II = 16% Astrocytoma grade II–III = 8% Astrocytoma grade III = 8% Ganglioglioma = 12% | 20 (74%)  VPS = 6 VAS = 3 ETV = 11 | GTR = 60% STR = 28% Bx = 12% | | 4 (15%) | 8 (30%) | | | At least 5/27 (19%) Clinical (duration to PD N/A) | 22/27 (81%) 5/27 (19%) died of PD | | Age 1–5 y = 11 y Age 5–10 y = 9 y Age >10 y = 5 y | | |
| Dabscheck et al.^7^ | 2015 | 66 | 10.7  (0.2–19.1) | | 35 (53%) | | JPA = 8 LGG = 4 Glioma with piloid features = 3 | 56 (85%)  VPS/ETV—no details | STR/Bx = 17 (25.8%)  No details | | N/A | N/A | | | N/A | N/A | | 7.7 y (1–24) | | |
| Aarsen et al.^1^ | 2014 | 12 | 10.2 (0.25–17.3) | | NA | | PA = 2 Neurocytoma = 1 Ganglioglioma = 1 | 12 (100%) ETV = 9; 1 converted to VPS VPS = 3 | 4 (33%)  Bx = 3 STR = 1 | | 0 | 0 | | | 1/12 (8%)  Clinical (duration to PD N/A) | 12/12 (100%) | | 2.75 y  (1–9.6 mos) | | |
| Griessenauer et al.^17^ | 2014 | 44 | 10.2  (mean)  (± 4.3) | | 27 (61%) | | PA = 3 Inconclusive = 2 | 36 (82%)  ETV = 19; 1 converted to VPS VPS = 17; 1 converted to ETV | 5 (11%) Bx = 4 (9%) Resection = 1 (2%) | | 3 (7%) | 2 (5%)  No details | | | 14/44 (32%) Radiographic (duration N/A) | 44/44 (100%) | | 7.9 y  (1.5–14.7) | | |
| Diaz et al.^9^ | 2014 | 14 | 16.5  (6.4–59) | | 9 (64%) | | N/A | 14 (100%)  ETV = 11 VPS → ETV = 3 | 0 | | 0 | 0 | | | 0 | 14/14 (100%) | | 3.9 y (2.2–7) | | |
| Ramelli et al.^37^ | 2011 | 10 | 12.5  (0.9–18) | | 6 (60%) | | N/A | 10 (100%) = ETV | 0 | | 0 | 0 | | | N/A | 10/10 (100%) | | 6 y (2.5–11) | | |
| Ternier et al.^43^ | 2006 | 40 | 9.4  (0–17.6) | | 12 (30%) | | LGG = 11 (including 5 PA) Dysplasia = 1 HGG = 1 Inconclusive = 1 | 37 (93%)  ETV = 27 (9 required another ETV at a mean of 6 mos) VPS = 10 (8 required shunt revision in first 3 y) | 14 (35%)  GTR = 4 STR = 10 | | 2 (5%) | 0 | | | 14/40 (35%) Radiologic (mean 20 mos [10 days–6.5 y]) | 39/40 (97.5%) 1 (2.5%) died of PD (HGG) | | 4.36 y (mean) | | |
| Li et al.^26^ | 2005 | 31 | 11  (mean) (0.12–20) | | 16 (52%) | | NA | 31 (100%)  VPS=23; 10 converted to ETV ETV = 8 | 0 | | 0 | 0 | | | N/A | 31/31 (100%) | | 8 y (mean) | | |
| Ramina et al.^38^ | 2005 | 7 | 13  (8–17) | | 6/8 (75%) | | LG astrocytoma = 7 | 7 (100%)  ETV = 6 VPS = 1 | 7 (100%)  GTR = 6 STR = 1 | | 2 (29%) | 0 | | | 1/7 (14%)  Clinical at 3 y | 6/7 (86%) 1 (14%) died of PD | | 9.9 y (mean) ± 5.9 | | |
| Stark et al.^42^ | 2005 | 12 | 6.75  (mean) (0.08-16y) | | 4 (33%) | | Astrocytoma = 4 (grade I = 1; grade II = 2; LG = 1) Ependymoma (grade II) = 1 | 12 (100%)  ETV = 5 VPS = 5 (4 required ETV for shunt failure) Resection + VPS = 2  5/9 ETV failure; 2 converted to VPS | 3 (25%) had resection | | 1 (8%) | 0 | | | 3/12 (25%) Radiologic at 6, 7, 12 y | 12/12 (100%) | | 9.5 y  (3 mos–28 y) | | |
| Javadpour and Mallucci^20^ | 2004 | 6 | 11.5  (9–19) | | 3 (50%) | | LG astrocytoma = 1 Inconclusive = 1 | ETV in 6 (100%); 2 converted to VPS | Bx = 2 (33%) | | NA | NA | | | 0 | 6/6 (100%) | | 31.5 mos  (20–45) | | |
| Dağlıoğlu et al.^8^ | 2003 | 9 | 12  (6–17) | | 5 (56%) | | NA | VPS = 9 (100%) | 0 | | 0 | 0 | | | 0 | 9/9 (100%) | | 5.3 y  (2.6–13.2) | | |
| Wellons et al.^48^ | 2002 | 13 | 10  (4–16) | | 8 (62%) | | NA | ETV = 13 (100%) | 0 | | 0 | 0 | | | 4/13 (31%) Radiographic (Duration N/A) | 13/13 (100%) | | 31 mos  (2–64) | | |
| Gómez-Gosálvez et al.^15^ | 2000 | 8 | 10  (0.3–16) | | 4 (50%) | | Inconclusive = 1 | 8 (100%)  VPS = 7 ETV = 1 | Bx = 1 (13%) | | 0 | 0 | | | 1/8 (13%) Radiographic (Duration N/A) | 8/8 (100%) | | 4 y  (9 mos–7 y) | | |
| Bowers et al.^4^ | 2000 | 7 | 6.3  (3.3–16.7) | 1 (14%) | | Consistent but not diagnostic of infiltrating non-pilocytic astrocytoma = 1 Negative for tumor = 2 | | VPS = 7 (100%) | Bx = 3 (43%) | 1 (14%) (56.8 Gy) | | 0 | | 6/7 (86%) Radiographic Median 0.95 y (0.3–5.7) | | | 7/7 (100%) | 3.8 y  (0.64–8.8) | | |
| Grant et al.^16^ | 1999 | 11 | 11  (5–14) | | 4 (36%) | | LGG = 1 | 11 (100%) VPS = 10 ETV = 1 | Bx = 1 (9%) | | 0 | 0 | | | 3/11 (27%) Radiographic  at 1,4,7 y | 11/11 (100%) | | 5 y (1–10) | | |
| Poussaint et al.^34^ | 1998 | 32 | 8 (mean) (0.17–17) | | 16 (50%) | | PA = 5 LGG = 2 Insufficient = 1 | 31 (96.9%)  VPS = 20 ETV = 7 Ventriculo-cervical shunting = 3 Cisternal-cervical shunting = 1 | 3 (9%)  Resection = 2 Bx = 1 | | 9 (28%)  median 54 Gy (52.5–56.2 Gy) | 0 | | | 10/32 (31%) Clinical Time from Dx to intervention for PD: mean 13 mos (2–40 mos) | 32/32 (100%) | | Observation group: 5 y (mean) (0.25–24) Intervention group: 5 y (mean) (0.25–17) | | |
| Pollack et al.^33^ | 1996 | 5 | 8  (4–11) | | 2 (40%) | | NA | VPS = 4 (80%) | N/A | | 0 | 0 | | | 1/5 (20%) Radiographic (2.17 y after Dx) | 5/5 (100%) | | 4 y (1–8) | | |
| Robertson et al.^39^ | 1995 | 8 | 10  (3–18) | | 5 (63%) | | NA | VPS = 7 (87.5%) | 0 | | 0 | 0 | | | 0 | 8/8 (100%) | | 3.08 y  (1.08–7.25) | | |
| Squires et al.^41^ | 1994 | 12 | 9.8 (mean)  (3–17) | | 7 (58%) | | Low-grade astrocytoma = 1 Inconclusive = 1 | VPS = 12 (100%) | Bx = 2 (17%) | | 3 (25%) | 2 (17%) (chemo-radiation) | | | 3/12 (25%) Clinical  + Radiologic (Duration N/A) | 12/12 (100%) | | Median =  4 y | | |
| Pollack et al.^32^ | 1994 | 16 | 9.75  (0.5–14) | | 9 (56%) | | Benign mixed glioma = 1 Anaplastic astrocytoma = 1 Low-grade astrocytoma = 1 | 16 (100%)  VPS = 14 ETV = 2; both converted to VPS | Bx = 3 (19%) | | 4 (25%) | 0 | | | 4/16 (25%) Clinical Median 7.8 y | 16/16 (100%) | | Median =  4.75 y  (0.75–21) | | |
| Lapras et al.^25^ | 1994 | 8 | 12  (8–17) | | 2/8 (25%) | | Juvenile type astrocytoma = 4 Astrocytoma grade I = 1 Astrocytoma grade II = 1 Astrocytoma grade III = 1 Oligodendroglioma= 1 | VPS = 8 (100%) | 8 (100%)  GTR = 5 Partial resection = 3 | | 2 (25%) | 2 (25%) | | | N/A | 8/8 (100%) | | 23.5 mos  (9–182) | | |
| Vandertop et al.^45^ | 1992 | 6 | 7.3  (2.5–15.75) | | 3 (50%) | | All non-pilocytic LG astrocytoma (6) | VPS = 5 (83%) | 6 (100%)  "Major" resection in 5 | | 2 (33%) | 0 | | | N/A | 6/6 (100%) | | 29.5 mos  (3–60) | | |
| Boydston et al.^5^ | 1991 | 6 | 15  (4–20) | | 5 (83%) | | PA = 4 | VPS = 6 (100%) | Bx = 4 (67%) | | 3 (50%) | 0 | | | 1/6 (17%) Clinical at 3 mos | 6/6 (100%) | | Range  2–10 y | | |
| May et al.^28^ | 1991 | 6 | 12.5  (5–17) | | 5 (83%) | | N/A | VPS = 6 (100%) | 0 | | 0 | 0 | | | 0/6 (0%) | 6/6 (100%) | | 5 y (8 mos–17 y) | | |

N, number; Dx, diagnosis; CSF, cerebrospinal fluid; Bx, biopsy; RGNT, rosette-forming glioneuronal tumor; RT, radiotherapy; PD, progressive disease; y, year(s); mos, months; PA, pilocytic astrocytoma; LGG, low-grade glioma; HGG, high-grade glioma; VPS, ventriculo-peritoneal shunt; ETV, endoscopic third ventriculostomy; GTR, gross total resection; NA, not applicable or data not available.
